# Unrestricted Feed Intake Induces β-Cell Death and Impairs Insulin Secretion in Broiler Breeder Hens

**DOI:** 10.3390/ani10111969

**Published:** 2020-10-26

**Authors:** Yu-Feng Huang, Ling-Chu Chang, Chung-Yu Chen, Yu-Hui Chen, Rosemary L. Walzem, Shuen-Ei Chen

**Affiliations:** 1 Department of Animal Science, National Chung Hsing University, Taichung 40227, Taiwan; husky_1016@hotmail.com (Y.-F.H.); katy0142002@hotmail.com (C.-Y.C.); cute5409@yahoo.com.tw (Y.-H.C.); 2 Chinese Medicinal Research and Development Center, China Medical University Hospital, Taichung 40447, Taiwan; t27602@mail.cmuh.org.tw; 3 Center for Molecular Medicine, China Medical University Hospital, Taichung 40447, Taiwan; 4 Department of Biological Science and Technology, China Medical University, Taichung 40447, Taiwan; 5 Department of Poultry Science, Texas A&M University, College Station, TX 77843, USA; rwalzem@tamu.edu; 6 The iEGG and Animal Biotechnology Center, National Chung Hsing University, Taichung 40227, Taiwan; 7 Research Center for Sustainable Energy and Nanotechnology, National Chung Hsing University, Taichung 40227, Taiwan; 8 Innovation and Development Center of Sustainable Agriculture (IDCSA), National Chung Hsing University, Taichung 40227, Taiwan

**Keywords:** broiler breeder hens, β-cells, apoptosis, insulin secretion, glucose disposal

## Abstract

**Simple Summary:**

Ad-feed intake caused transient hyperinsulinemia, but ultimately impaired insulin secretion and glucose clearance leading to hyperglycemia in broiler breeder hens. The impairments were operated at insulin gene expression and at pyruvate anaplerosis for ATP supply for insulin release. Lipotoxicity, inflammation, and cell apoptosis in the β-islets contributed to impaired insulin secretion in Ad-hens.

**Abstract:**

Past studies regarding to insulin secretion and glucose disposal in chickens were focused on rapidly growing juvenile broilers and may not reflect glucose/insulin physiology in adulthood. The study aimed to assess insulin secretion and glucose disposal in respect to restricted (R) vs. ad libitum (Ad) feed intake for obesity development in broiler breeder hens. Hens at age of 26 weeks were continued on R rations, or allowed Ad-feed intake up to 45 weeks. Results from prandial changes and glucose tolerance test suggested that Ad-feed intake to 45 weeks impaired insulin secretion and glucose clearance, and, thus, caused hyperglycemia in accompany with transient hyperinsulinemia at age of 33 weeks (*p* < 0.05). The alterations were shown operating at both transcript and protein level of insulin gene expression per se and at ATP supply for insulin release as evidenced by consistent changes of enzyme expression and activity in pyruvate anaplerosis in the β-islets (*p* < 0.05). Ad-feed intake also increased β-islet triacylglycerol and ceramide accumulation and provoked interleukin-1β (IL-1β) production (*p* < 0.05), which were further manifested by a detrimental increase of caspase 3/7 activity and cell apoptosis (*p* < 0.05). Results support the conclusion that release to Ad-feed intake in broiler breeder hens transiently induced hyperinsulinemia along rapid bodyweight gain and adiposity, but later provoked lipotoxicity and inflammation leading to β-cell apoptosis and ultimately impaired insulin secretion and glucose disposal.

## 1. Introduction

Modern broilers are the products of long-term genetic selection for early rapid growth. However, such selection strategies also resulted in a tendency to overeat and several collateral defects such as obesity, ascites, and poor reproductive performance [1,2,3,4]. Alterations in glucose disposal, insulin secretion, and sensitivity are often invoked to explain phenotypic differences arising from genetic selection for rapid vs. slow growth and fatness vs. leanness or dwarf vs. normal broilers [5,6,7,8].

Compared to mammals, granivorous avian species such as chickens maintain elevated fasting blood glucose without an overt diabetes [7,9]. Insulin secretion in birds is relatively insensitive to glucose stimulation [9]. Indeed, while culturing or perfusing rodent islets, a dramatic biphasic insulin secretion was prompted by glucose at 8.5–11.1 mM [10], however, as high as 30–40 mM glucose induced only a modest increase of insulin secretion in isolated and perfused chicken pancreas [9,11]. To date most studies of glucose disposal and insulin secretion were carried out using growing juvenile broilers, often before 6 weeks of age during a period of massive lean tissue gain. It remains uncertain whether the results of these studies appropriately reflect glucose/insulin physiology in adulthood. 

Broiler breeder hens are severely feed restricted to improve livability and egg production [1,3,4,12,13]. However, this approach is under increasing scrutiny due to the concern of undue hunger for animal welfare. More scientific studies are required to address the genetic defects of current broiler chickens and provide objective evidences in the issue regarding to animal productivity vs. animal welfare. Comprehensive reports of insulin secretion and glucose disposal in adult chickens remain elusive. Information on this aspect of physiology may prove particularly important in broiler breeder hens that are obesity prone and susceptible to co-morbid metabolic derangements that contribute to ascites and cardiomyopathy [2,14,15]. We sought to characterize insulin secretion and glucose disposal in broiler breeder hens reared to the point of lay to achieve breeder company target bodyweights for age. Pancreatic β-islets from feed-restricted (R) hens or hens allowed to consume feed to appetite (ad libitum (Ad)) were used for molecular, biochemical, and functional studies for the mechanisms underlying the changes of insulin secretion.

## 2. Materials and Methods

### 2.1. Animal Management

A flock of broiler breeder hens (Arbor Acres Plus FF) at age 23 weeks were purchased from a local breeder farm. Hens were caged individually and fed a standard soy and corn-based breeder layer mash (Supplementary Table S1) with weekly adjustment of feed allocation to achieve the targeted bodyweight as recommended (Supplementary Table S2). Birds were maintained on a 14 L:10 D photoschedule (lights on at 05:00 a.m.) with feed placed at 08:30 a.m. Water was available at all times. Egg production and feed intake were recorded daily. Starting at age 26 weeks, half of the birds (*n* = 70) were continued with breeder recommended feed restriction (R-group) while remaining hens were provided with sufficient feed for consumption to appetite (Ad-group). All bird husbandry and tissue collections were in accordance with an approved animal care protocol (IACUC Permit NO. 99–74) in the National Chung University, Taiwan.

### 2.2. Necropsy and Tissue Collection

Four 26 week-old R-hens were used to establish baseline body composition values. When hens were 33 and 45 weeks old, 6 hens from each group were randomly selected for necropsy for tissue collection. Collected liver and adipose tissue were weighed for composition analysis. A caudal portion (1/4 in the total length) of the dorsal lobes of the pancreas was excised and used for immunohistochemical and in situ apoptosis analysis. A separate portion was used to determine insulin gene expression by qRT-PCR and protein amounts by Western blot. Residual pancreas was used for β-cell isolation [16]. Isolated β-cells were used for biochemical, enzyme activity, and gene expression analyses.

### 2.3. Prandial Changes of Plasma Insulin and Glucose Levels

Two days before necropsy for tissue collection, the selected 6 hens from each group were fasted for 12 h, and then re-fed with 30 g of feed. Fasted blood and 30 min postprandial blood samples were drawn into citrate tubes, kept on ice, and centrifuged (2500× *g* at 16 °C for 15 min) within 30 min. Plasma glucose concentration was measured enzymatically using a commercial kit (Wako, Osaka, Japan). Plasma insulin levels were determined by a validated commercial ELISA kit (Cat. # 10-1249-01, Uppsala, Sweden) [17].

### 2.4. Glucose Tolerance Test

A separate set of additional 6 hens each from the R-hen and Ad-hen groups were randomly selected at 33 and 45 weeks for glucose tolerance testing. Twelve hour fasted hens were cannulated at the wing vein using a SafeTouch Safety Winged Infusion Set (23 g × 3/4, 12″ Tubing, Nipro Corporation, Osaka, Japan). A bolus of glucose (0.5 g/mL in saline, 2 mL/kg bodyweight) was manually infused at a rate 4 mL/min following a zero time blood sampling. Timed blood samples were collected at 5, 15, 30, 60, 90, and 120 min after glucose infusion.

### 2.5. Isolation of Pancreatic β-Islets

Isolation of pancreatic β-islets was performed with modifications according to the method from Datar and Bhonde [16]. Briefly, finely minced pancreas (approximately in 0.3 cm^3^) were incubated with collagenase-type V (1.5 mg/mL, Sigma-Aldrich, St. Louis, MO, USA) in Hanks’ balanced salt solution (HBSS) at 37 °C for 5 min. After a slow centrifugation (50× *g*, 2 min), the supernatant was collected and kept on ice and the pellets were subjected to an additional round of digestion at 37 °C for 5 min. These steps were repeated twice. Digestion was stopped by addition of cold HBSS with 1% fetal calf serum (FBS). Collected supernatants were combined and filtered through nylon mesh filter with 250 μm in pore size. Cold HBSS was pored over the filter to wash remaining islets through. Filtered supernatants were held on ice for 20 min to allow islets to settle prior to decanting approximately 90% of supernatant. Islets were contained in the bottom 10% of the total volume, and were collected following centrifugation at 60× *g* for 3 min, 4 °C. An aliquot of the pellets was stained with diathizone to confirm the purity of isolated β-islets [18] (Supplementary Figure S1). The pellets were stored at –80 °C until use.

### 2.6. Immunohistochemistry and Apoptosis Analysis

A caudal portion (1/4 in the total length) of the dorsal lobes of pancreas was fixed in 4% formaldehyde and embedded in paraffin. Following sectioning, embedded pancreas sections were de-waxing for antigen-retrieval as described previously [19]. De-waxed pancreatic sections were then incubated with a validated rabbit anti-bovine/porcine insulin antibody at 4 °C overnight (1:200 in 1% BSA of PBS-0.01%Tween 20) (InnunoStar Inc., Lot#1317001, Hudson, WI, USA) [20]. In situ apoptosis analysis using a terminal transferase dUTP nick end labeling (TUNEL) assay kit (Click-iT^®^ Plus, Roche Applied Science, Indianapolis, IN, USA) was performed on the sections following several washes (PBS-0.01%Tween 20). After 2 times 10 min washes, the sections were incubated with a secondary antibody (goat anti-rabbit IgG conjugated with Alexa-467, in 1% BSA of PBS-0.01%Tween 20) at room temperature for 1 h and then washed prior to imaging. Three sections per hen, and 5 images per section were used to generate a mean value for fluorescence intensity quantification using Image-J software.

Protein extracts from freshly collected β-islet homogenates were used for caspase-3/7 activity analysis as determined by the cleavage rate of the fluorophoric peptide substrate (Caspase-Glo^®^ 3/7 assay system, Promega, Madison, WI, USA) according to the manufacturer’s instructions. The reaction was monitored up to 1.5 h using a microplate reader (Infinite F200 PRO, Tecan Group Ltd., Männedorf, Switzerland) with excitation/emission at 480/520 nm. Results were expressed at cumulative counts per minute (CPM).

### 2.7. Pancreatic β-Islet TG, Ceramide, MDA Content, SOD and PDH Activity Analysis

Pancreatic islet triacylglycerol (TG) content was determined following TG separation by thin layer chromatography and fatty acid quantification by gas chromatography as described previously [19]. Ceramide content was determined by measuring fluorescence of products formed following alkaline hydrolysis as described previously [19,21]. Superoxide dismutase (SOD) activity and malondialdehyde (MDA) content were determined according to the instructions enclosed in commercial kits (Item # 706002, # 708070, Cayman Chemical Company, Ann Arbor, MI, USA), respectively. Pyruvate dehydrogenase (PDH) activity was determined using a colorimetric kit (Cat. # K679-100, BioVision, Inc., Milpitas, CA, USA) [15].

### 2.8. Gene Expression by qRT-PCR Analysis

Sample preservation, total RNA extraction, random priming reverse transcription, and qRT-PCR amplification were conducted as described previously [19] using commercial kits (Applied Biosystems, Waltham, MA, USA). Supplementary Table S3 contains primer information. Reactions were conducted in triplicate and the intra-assay coefficient of variation (CV) was less than 10 %.

### 2.9. Western Blot Analysis

Western blot analysis was conducted as described previously [21,22] using a rabbit anti-bovine/porcine insulin polyclonal antibody (Lot#1317001, InnunoStar Inc.), anti-chicken interleukin-1β (IL-1β) (Cat. # ab-24771, Abcam, Cambridge, UK), anti-mouse AMP-activated protein kinase α subunit (AMPKα) (Cat. # 07-350, EMD Millipore Chemicals, Billerica, MA, USA), pyruvate kinase (PK) isozymes M1/M2 (Cat. #3186), and phospho-AMPKα (Thr172, Cat. # 2531), and anti-β-actin antibody (Cat. # 4967, Cell Signaling Technology, Danvers, MA, USA). A horseradish peroxidase-conjugated secondary antibody was used to identify the bands reactive to the primary antibodies through an enhanced chemiluminescence method (Pierce Biotechnology Inc., Rockford, IL, USA).

### 2.10. Statistics

Data were analyzed by two-way ANOVA, in which feed intake level (R or Ad) and prandial status (fasted or re-fed) or feeding duration (7 and 19 weeks in hens fed to 33 and 45 weeks of age, respectively) were the classifying variables. Differences between group means were tested using Bonferroni corrected *t*-test when the main effect was significant. If an interaction between feed intake and age was found, a mean comparison was performed. Values were expressed as means ± SEM. Mean differences were considered significant at *p* < 0.05 (*n* = 6 chickens). All statistical procedures were carried out using SPSS for Windows 13.0 (SPSS Inc., Chicago, IL, USA).

## 3. Results

### 3.1. Prandial Changes of Plasma Glucose and Insulin Levels

Release to Ad-feed intake resulted in a burst of feed consumption in the first week from the prescribed 145 to 216 g/day/hen (Supplementary Figure S2, panel A). Feed intake then declined gradually to below the breeder recommended amount (162 g/day/hen) after 34 weeks, and reached a nadir at 40 weeks (133 g/day/hen). The Ad-hens then increased feed intake to 157 g/day/hen at 45 weeks. This pattern of feed intake was in marked contrast to a slow increase in feed intake of R-hens recommended by the breeder company to control targeted BW gains from ~3.13 kg at 26 weeks to ~3.55 kg at 45 weeks. The BW of Ad-hens increased sharply to reach 4.33 kg/hen at age of 36 weeks, declined slightly to 4.2 kg at 40 weeks, and, subsequently, increased to reach 4.35 kg/hen at 45 weeks (Supplementary Figure S2, panel B). Ad-hens also exhibited a consistently lower egg production rate after 31 weeks (Supplementary Figure S2, panel C). In addition to a rapid BW gain, relative weights (g/100 g BW) of both abdominal adipose and liver were increased in Ad-hens at 33 and 45 weeks (*p* < 0.05, Supplementary Figure S2, panel D). The increase in relative adipose and liver weights was rapid with the greatest increase happening from 26 to 33 weeks. 

**Figure 1:**
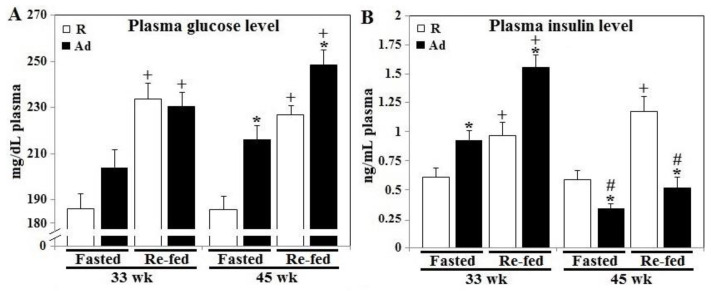
Prandial changes of plasma glucose and insulin levels of broiler breeder hens provided with restricted or ad libitum feed intake. Two days before necropsy for tissue collection at 33 and 45 weeks, blood samples were collected from the selected hens after overnight fasting and 30 min after re-feeding (*n* = 6). Plasma glucose (panel **A**) and insulin (panel **B**) concentrations were measured. *; significant difference by Ad-feed intake (*p* < 0.05). +; significant difference by re-feeding (*p* < 0.05). #; significant difference vs. corresponding hens sampled at 33 week (*p* < 0.05). R; restriction, Ad; ad libitum.

Fasting plasma glucose levels in R-hens were similar at week 33 and 45, being ~185 mg glucose/dL plasma (Figure 1, panel A). R-hen postprandial glucose response to feed allowance was also similar at 33 and 45 weeks with a 27% (50 mg glucose/dL) increase above fasting values (*p* < 0.05). Ad-feed intake increased fasting and postprandial plasma glucose concentrations at 45 weeks, being 218 mg glucose/dL and 250 mg glucose/dL, respectively, and the postprandial glucose value increased an average of 13% at both 33 and 45 weeks in Ad-hens (*p* < 0.05, Figure 1, panel A). Changes in plasma insulin concentration occurred earlier, following Ad feed intake as both fasted and postprandial insulin concentrations in Ad-hens were significantly increased at 33 weeks (by ~40% and 75%, respectively) compared to R-hens (*p* < 0.05, Figure 1, panel B). Interestingly, while 45 week fasting and postprandial plasma insulin concentrations in R-hens were similar to 33 week values, both values in Ad-hens were significantly lower compared to either Ad-hen 33 week (by ~60% and 68%) or R-hen values at 45 weeks (by ~40% and 48%, respectively) (*p* < 0.05). Plasma insulin responses in Ad-hens at 45 weeks perhaps reflect β-cell exhaustion and impaired secretion (Figure 1, panel B).

### 3.2. Glucose Disposal, Insulin Secretion, and Gene Expression

**Figure 2:**
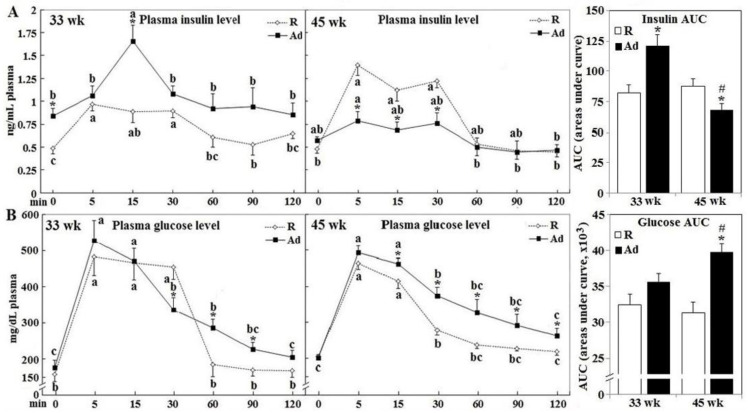
Insulin secretion and glucose disposal of broiler breeder hens provided with restricted or ad libitum feed intake. At age of 33 and 45 weeks, 6 overnight fasted hens from each the R- and Ad-groups were cannulated at the wing vein and given a glucose (0.5 g/mL, 2 mL/kg bodyweight) bolus (4 mL/min infusion rate). Timed blood samples were collected and analyzed for insulin (panel **A**) and glucose (panel **B**) concentration (*n* = 6). *; significant difference by Ad-feed intake (*p* < 0.05). #; significant difference vs. 33 weeks (*p* < 0.05). Means with different letters within the same feed intake are significantly different among times (*p* < 0.05). R; restriction, Ad; ad libitum, AUC; areas under curve.

**Figure 3:**
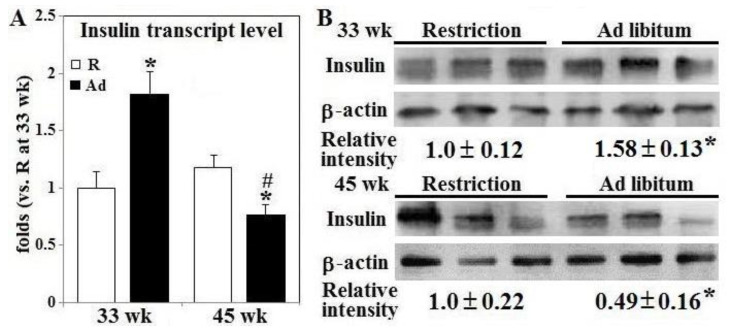
Insulin gene expression in the pancreas of broiler breeder hens provided with restricted or ad libitum feed intake. Insulin gene expression (panel **A**, qRT-PCR) and protein amount (panel **B**, Western blot) were determined in the pancreas from hens necropsied at age of 33 and 45 weeks (*n* = 6). Results of qRT-PCR and Western blot study were normalized to β-actin and expressed as ratios relative to R-hens at 33 or 45 weeks. *; significant difference by Ad-feed intake (*p* < 0.05). #; significant difference vs. 33 weeks (*p* < 0.05). R; restriction, Ad; ad libitum.

Intravenous glucose tolerance testing was used to further confirm impaired insulin secretion and glucose disposal following Ad-feed intake. In 33 week-old R-hens, insulin concentration increased from 0.5 ng/mL to its peak of 0.9 ng/mL in the first 5 min post glucose infusion and gradually returned to near baseline by 60 min (*p* < 0.05, Figure 2, panel A), coincident with return of plasma glucose to fasting concentrations (Figure 2, panel B). A similar insulin response pattern was observed in 45-week old R-hens, however the peak concentration was 44% higher being 1.3 ng/mL; insulin returned to fasting level at 60 min in accompany with plasma glucose concentration (*p* < 0.05, Figure 2, panels A,B). Insulin response to glucose infusion differed in Ad-hens and was influenced by the duration of Ad-feed intake. Ad-hens exhibited fasting hyperinsulinemia at 33 weeks, with plasma concentrations similar to the 5 min peak value of R-hens, followed by a dramatic rise to 1.6 ng/mL at 15 min, nearly twice the peak concentration of R-hens of the same time, and after plasma glucose concentration peak at 5 min (*p* < 0.05, Figure 2, panel A). Ad-hens failed to show an insulin response to glucose infusion at 45 weeks leading to a lower total area under the curve (AUC) for insulin of the 120 min test (*p* < 0.05, Figure 2, panel A). At both 33 weeks and 45 weeks, R-hens glucose concentrations peaked at 5 min and returned to fasting levels by 60 min post infusion (*p* < 0.05, Figure 2, panel B). In Ad-hens, plasma glucose levels also peaked at 5 min but showed a delayed return to baseline concentrations, such that glucose AUC was elevated relative to R-hens at both 33 weeks (10%) and 45 weeks (25%) (Figure 2, panel B). As a result, Ad-hens had significantly higher insulin AUC but no change of glucose AUC at 33 weeks, and lower insulin AUC but higher glucose AUC at 45 weeks (*p* < 0.05, Figure 2, panel A,B). However, the pattern and magnitude of glucose clearance in Ad-hens was similar at 33 weeks and 45 weeks, indicating little linkage to circulating insulin per se. In consistence with changes of insulin secretion following glucose infusion or during the fasted-re-feeding transition, Ad-feed intake transiently promoted insulin gene expression at both transcript and protein levels but impaired its expression at 45 weeks (*p* < 0.05, Figure 3, panels A,B).

### 3.3. AMPK Activation and Pyruvate Metabolism

**Figure 4:**
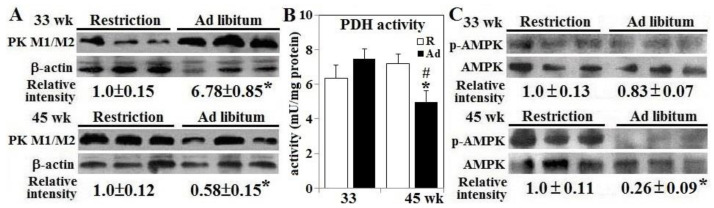
Activation of AMP-activated protein kinase and pyruvate kinase expression in the pancreatic β-islets of broiler breeder hens provided with restricted or ad libitum feed intake. Isolated β-islets from hens necropsied at age of 33 and 45 weeks were used for pyruvate kinase (PK) M1/M2 expression (panels **A**,**B**) and AMP-activated protein kinase (AMPK) activation (panel **C**) through Western blot method and pyruvate dehydrogenase (PDH) activity by a calorimetrical method (*n* = 6). Results of Western blots were normalized to pan form of AMPK (unphosphorylated) or β-actin and expressed as ratios relative to R-hens. *; significant difference by Ad-feed intake (*p* < 0.05). #; significant difference vs. 33 weeks (*p* < 0.05). R; restriction, Ad; ad libitum.

Ad-feed intake significantly increased β-islet PK expression up to 6.8 folds at 33 weeks and suppressed the expression by 42% at 45 weeks (*p* < 0.05, Figure 4, panel A). Interestingly, PDH activity and AMPK activation remained unchanged at 33 weeks but PDH activity was decreased by 30% in Ad-hens at 45 weeks in conjunction with 74% decrease in AMPK activation (*p* < 0.05, Figure 4, panels B,C). 

### 3.4. Lipotoxicity, Oxidative Stress, Inflammation Status, and β-Cell Apoptosis

**Figure 5:**
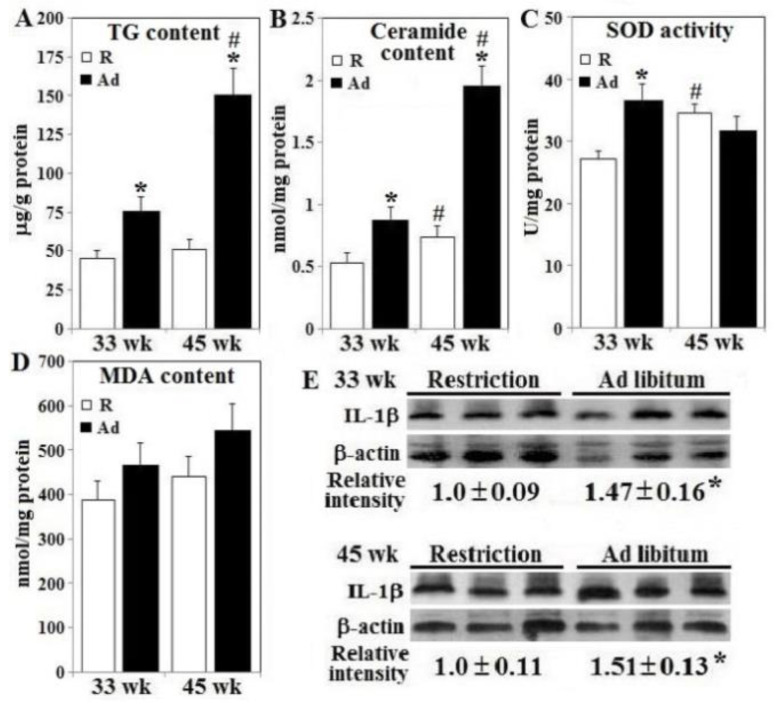
Lipotoxic development, oxidative stress and inflammation in the pancreatic β-islets of broiler breeder hens provided with restricted or ad libitum feed intake. Isolated β-islets from hens necropsied at age of 33 and 45 weeks were used for triacylglycerol (TG, panel **A**), ceramide content (panel **B**), superoxide dismutase (SOD) activity (panel **C**), MDA malondialdehyde (MDA) content (panel **D**), and interleukin-1β (IL-1β) expression (panel **E**) analysis (*n* = 6). Result of Western blots (panel **E**) were normalized to β-actin and expressed as ratios relative to R-hens. *; significant difference by Ad-feed intake (*p* < 0.05). #; significant difference vs. 33 weeks (*p* < 0.05). R; restriction, Ad; ad libitum.

**Figure 6:**
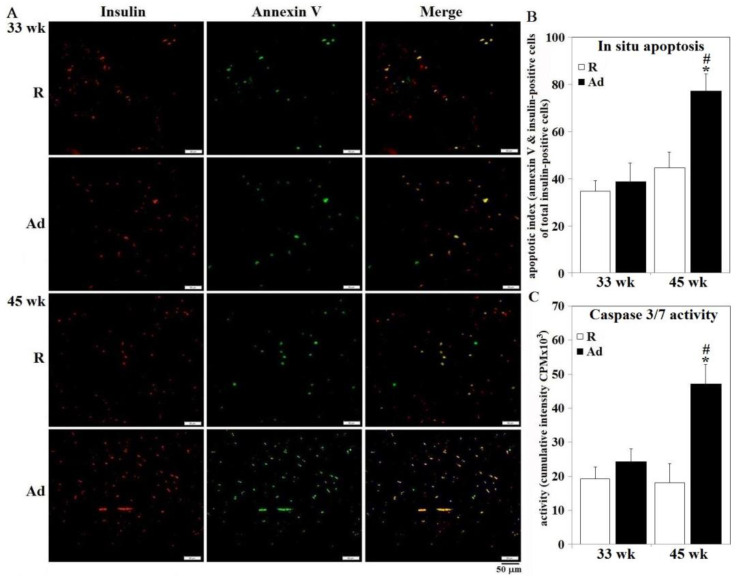
Pancreatic β-cell apoptosis of broiler breeder hens provided with restricted or ad libitum feed intake. Isolated β-islets from hens necropsied at age of 33 and 45 weeks were used for immunohistochemical staining by insulin antibody followed by TUNEL labeling for in situ apoptosis of β-cells (panel **A**) and for caspase 3/7 activity analysis (panel **C**) using a fluorophoric peptide substrate (*n* = 6). Results of apoptosis were quantified by Image-J software (panel **B**). *; significant difference by Ad-feed intake (*p* < 0.05). #; significant difference vs. 35 weeks (*p* < 0.05). R; restriction, Ad; ad libitum.

Pancreatic β-islets from Ad-hens had elevated concentrations of TG, ceramide, and increased IL-1β production at both 33 weeks and 45 weeks compared to R-hens, with effects greatest at 45 weeks (Figure 5, panels A,B,E). Superoxide dismutase activity in Ad-hens at 33 weeks was greater than that of R-hens and R-hen SOD activity increased from 33 to 45 weeks (*p* < 0.05), whereas MDA concentrations in β-islets were similar in both groups at both time points (Figure 5, panels C,D). In accordance with impaired insulin gene expression and secretion, prolonged Ad-feed intake to 45 weeks induced β-cell apoptosis in conjunction with increased caspase 3/7 activity (Figure 6, panels A,B, supplementary Figure S3).

## 4. Discussion

We have repeatedly demonstrated that prolonged Ad-feed intake by broiler breeder hens produces lipotoxicity and inflammatory response arising from lipid dysregulation and associated changes in gene expression and signaling that results in impaired function in ovarian follicles, circulating leukocytes and heart [14,15,19,21]. We now demonstrate that tissue dysfunction extends to pancreatic β-cells, compromising insulin secretion and glucose metabolism.

Hyperglycemia and impaired glucose disposal following prolonged Ad-feed intake by broiler breeder hens can be partially attributed to impaired insulin secretion due to a loss of β-cells through apoptosis arising from lipotoxicity. Ceramide accumulation impairs insulin secretion by suppressing nuclear translocation of pancreatic and duodenal homeobox 1 (PDX-1) and musculoaponeurotic fibrosarcoma oncogene homologue A (MafA) expression, the two critical transcription factors in insulin gene expression [23]. Suppression of PDX-1 and MafA due to excessive reactive oxygen species (ROS) also impairs insulin secretion during high glucose challenges [23]. Accordingly, lipotoxic development and inflammatory conditions due to ceramide signaling and TG accumulation were concluded to contribute to β-cell apoptosis leading to impaired insulin secretion and gene expression in Ad-hens.

In most of mammalian tissues, converting pyruvate to acetyl-CoA by PDH to enter Krebs cycle in the mitochondria is a critical step in glucose oxidation for ATP generation [24]. Insulin secretion requires intracellular ATP to trigger the closure of ATP-sensitive K^+^ ion channels and the following events for insulin release. In contrast to other tissues, β-islets are relatively sensitive in pyruvate oxidation; prolonged exposure to high levels of glucose or fatty acids impairs PDH activity in the β-islets [25,26]. However, pancreatic β-cells possess a unique defense to ensure glucose oxidation for sufficient ATP supply for insulin secretion. The unique defense is achieved through pyruvate anaplerosis pathway comprising pyruvate carboxylase (PC), phosphoenolpyruvate carboxykinase (PEPCK), and PK action for pyruvate and oxaloacetate (OAA) production and fuel influx/efflux through the pyruvate malate shuttle on the mitochondrial membranes. Accordingly, the unique response in β-islets is operative in hens as evidenced by dramatic upregulation of PK in Ad-hens at 33 weeks without a change in PDH activity, AMPK activation, and cell survival and, thus, may account for the transient hyperinsulinemia by Ad-feed intake. However, cytosolic accumulation of pyruvate and OAA may promote ATP-citrate lyase activity leading to increased acetyl-CoA concentrations, and, thereby, enhance fatty acid and TG synthesis in favor of lipotoxic and inflammatory development in the β-cells [23,25,26]. 

Most of past studies regarding to insulin secretion and glucose disposal in chickens were conducted in growing juvenile broilers. Results of those showed, for example, that in contrast to a slow growth strain, a rapid juvenile growth strain of broilers exhibited higher fasting insulin levels but slightly lower fasting plasma glucose levels, and a similar glucose disposal rate; albeit with a higher glucose-induced insulin secretion [7]. Genetically fat broilers had slightly higher plasma insulin levels but lower glucose levels, and a faster glucose disposal rate and higher sensitivity than their lean counterparts [9,27]. In Dwarf broiler chickens, however, except for higher insulin sensitivity, no differences in plasma insulin and glucose levels, and glucose disposal was noted, when compared with normal broilers [9]. In the study, birds in the latter stage (after 35 weeks) had a much slower BW gain and fattening compared to early stage, thus the difference of insulin secretion and action, as well as glucose disposal in different ages (33 vs. 45 weeks) are more much like those across strains such as fast vs. slow growth or fat vs. lean line. When allowed Ad-feed intake, a systemic resistance to insulin action may occur as birds approach the genetically-targeted BW and adiposity in the later stage. 

Studies with juvenile broilers suggested that only the liver responds to insulin stimulation, whereas muscle had a dramatic activation of early Akt signaling, but not in downstream cascades and adipose tissue was unresponsive to insulin stimulation in Akt signaling [6,28,29]. In fed juvenile broilers, immuno-neutralization of circulating insulin decreased Akt activation in both liver and muscle, but not in adipose tissue [6], suggesting that the liver and muscle are responsive to endogenous insulin. Interestingly, a blockade of circulating insulin increased plasma glucose levels but suppressed feed intake independent of 23 marker gene expressions of hunger and satiety regulation in the hypothalamus [30]. Thus, upon release to Ad-feed intake, transient hyperinsulinemia and enhanced insulin secretion in the early stage may facilitate fuel partitioning toward the liver and muscular tissues such as the heart, and, thereby, exacerbate pathogenic progression leading to fatty liver steatosis and metabolic cardiomyopathy, and finally compromised cardiac function [12]. Further studies to specifically examine insulin actions particularly on glucose uptake by specific organs or tissues in conjunction with changes in body composition are under investigation. 

The present results first reported impaired insulin secretion and glucose disposal due to lipotoxicity and inflammation in the β-islets along rapid bodyweight gain and obesity development in broiler breeder hens provided with Ad-feed intake. Transit hyperinsulinemia and enhanced glucose-induced insulin secretion by Ad-feed intake may accelerate hepatic and cardiac pathogenic progression. Restricted feed intake results in slow BW gain to avoid metabolic derangements. 

## 5. Conclusions

Prolonged Ad-feed intake caused transient hyperinsulinemia, but ultimately impaired insulin secretion and glucose clearance leading to hyperglycemia in broiler breeder hens. Impaired insulin secretion by Ad-feed intake was operated at insulin gene expression and at pyruvate anaplerosis for ATP supply for insulin release. Lipotoxicity, inflammation, and cell apoptosis in the β-islets contribute to impaired secretion and glucose clearance disposal in Ad-hens.

## Supplementary Materials

The following are available online at https://www.mdpi.com/2076-2615/10/11/1969/s1, Table S1: Ingredient composition of the basal broiler breeder layer diet, Table S2: Recommended body weight and feed allowance of Arbor Acres Plus FF broiler breeder hens from age of 23 to 45 weeks., Table S3: Primers for quantitative real time polymerase chain reaction (qRT-PCR) analysis, Figure S1: Isolated β-islets of broiler breeder hens by diathizone staining, Figure S2. Body weight, feed intake, egg production, and fractional liver and abdominal fat weight of broiler breeder hens provided with restricted or ad libitum feed intake, Figure S3. Pancreatic β-islet caspase 3/7 activity of broiler breeder hens provided with restricted or ad libitum feed intake.

## References

[1] Griffin H.D., Goddard C. (1994). Rapidly growing broiler (meat-type) chickens: Their origin and use for comparative studies of the regulation of growth. Int. J. Biochem..

[2] Julian R.J. (2000). Physiological, management and environmental triggers of the ascites syndrome: A review. Avian Pathol..

[3] Chen S.E., McMurtry J.P., Walzem R.L. (2006). Overfeeding-induced ovarian dysfunction in broiler breeder hens is associated with lipotoxicity. Poult. Sci..

[4] Richards M.P., Proszkowiec-Weglarz M. (2007). Mechanisms regulating feed intake, energy expenditure, and body weight in poultry. Poult. Sci..

[5] Dupont J., Chen J., Derouet M., Simon J., Leclercq B., Taouis M. (1999). Metabolic differences between genetically lean and fat chickens are partly attributed to the alteration of insulin signaling in liver. J. Nutr..

[6] Dupont J., Tesseraud S., Derouet M., Collin A., Rideau N., Crochet S., Godet E., Cailleau-Audouin E., Métayer-Coustard S., Duclos M.J. (2008). Insulin immuno-neutralization in chicken: Effects on insulin signaling and gene expression in liver and muscle. J. Endocrinol..

[7] Dupont J., Scanes C.G. (2015). Endocrine Pancreas. Sturkie’s Avian Physiology Chapter 27-Endocrine Pancreas.

[8] Shiraishi J., Yanagita K., Fukumori R., Sugino T., Fujita M., Kawakami S., McMurtry J.P., Bungo T. (2011). Comparisons of insulin related parameters in commercial-type chicks: Evidence for insulin resistance in broiler chicks. Physiol. Behav..

[9] Rideau N., Harvey S., Etches R.J. (1997). Insulin secretion. Perspectives in Avian Endocrinology.

[10] Zawalich W.S., Yamazaki H., Zawalich K.C. (2008). Biphasic insulin secretion from freshly isolated or cultured, perifused rodent islets: Comparative studies with rats and mice. Metabolism.

[11] King D.L., Hazelwood R.L. (1976). Regulation of avian insulin secretion by isolated perfused chicken pancreas. Am. J. Physiol..

[12] Lin H.Y., Chung T.K., Chen Y.H., Walzem R.L., Chen S.E. (2019). Dietary supplementation of 25-hydroxycholecalciferol improves livability in broiler breeder hens. Poult. Sci..

[13] Lin H.Y., Chou P.C., Chen Y.H., Chung T.K., Lai L.S., Walzem R.L., Huang S.Y., Chen S.E. (2019). Dietary supplementation of 25-hydroxycholecalciferol improves livability in broiler breeder hens-amelioration of cardiac pathogenesis and hepatopathology. Animals.

[14] Chen C.Y., Lin H.Y., Chen Y.W., Ko Y.J., Liu Y.J., Chen Y.H., Walzem R.L., Chen S.E. (2017). Obesity-associated cardiac pathogenesis in broiler breeder hens: Pathological adaption of cardiac hypertrophy. Poult. Sci..

[15] Chen C.Y., Huang Y.F., Ko Y.J., Liu Y.J., Chen Y.H., Walzem R.L., Chen S.E. (2017). Obesity-associated cardiac pathogenesis in broiler breeder hens: Development of metabolic cardiomyopathy. Poult. Sci..

[16] Datar S.P., Bhonde R.R. (2010). Cryopreservation of chick islets. CryoLetters.

[17] Franssens L., Lesuisse J., Wang Y., De Ketelaere B., Willems E., Koppenol A., Guo X., Buyse J., Decuypere E., Everaert N. (2015). Prenatal tolbutamide treatment alters plasma glucose and insulin concentrations and negatively affects the postnatal performance of chickens. Domest. Anim. Endocrinol..

[18] Datar S.P., Suryavanshi D.S., Bhonde R.R. (2006). Chick pancreatic B islets as an alternative in vitro model for screening insulin secretagogues. Poult. Sci..

[19] Liu Z.C., Xie Y.L., Chang C.J., Su C.M., Chen Y.H., Huang S.Y., Walzem R.L., Chen S.E. (2014). Feed intake alters immune cell functions and ovarian infiltration in broiler hens-implications for reproductive performance. Biol. Reprod..

[20] Alarcon C., Serna J., Perez-Villamil B., de Pablo F. (1998). Synthesis and differentially regulated processing of proinsulin in developing chick pancreas, liver and neuroretina. FEBS Lett..

[21] Pan Y.E., Liu Z.C., Chang C.J., Xie Y.L., Chen C.Y., Chen C.F., Walzem R.L., Chen S.E. (2012). Ceramide accumulation and upregulation of proinflammatory interleukin-1β exemplify lipotoxicity to mediate declines of reproductive efficacy of broiler hens. Domest. Anim. Endocrinol..

[22] He X., Dagan A., Gatt S., Schuchman E.H. (2005). Simultaneous quantitative analysis of ceramide and sphingosine in mouse blood by naphthalene-2, 3-dicarboxyaldehyde derivatization after hydrolysis with ceramidase. Anal. Biochem..

[23] Poitout V., Hagman D., Stein R., Artner I., Robertson R.P., Harmon J.S. (2006). Regulation of the insulin gene by glucose and fatty acids. J. Nutr..

[24] Randle P.J., Priestman D.A., Mistry S., Halsall A. (1994). Mechanisms modifying glucose oxidation in diabetes mellitus. Diabetologia.

[25] Liu Y.Q., Tornheim K., Leahy J.L. (1999). Glucose-fatty acid cycle to inhibit glucose utilization and oxidation is not operative in fatty acid-cultured islets. Diabetes.

[26] Liu Y.Q., Moibi J.A., Leahy J.L. (2004). Chronic high glucose lowers pyruvate dehydrogenase activity in islets through enhanced production of long chain acyl-CoA: Prevention of impaired glucose oxidation by enhanced pyruvate recycling through the malate-pyruvate shuttle. J. Biol. Chem..

[27] Leclercq B., Whitehead C.C. (1998). Leanness in Domestic Birds.

[28] Dupont J., Dagou C., Derouet M., Simon J., Taouis M. (2004). Early steps of insulin receptor signaling in chicken and rat: Apparent refractoriness in chicken muscle. Domest. Anim. Endocrinol..

[29] Dupont J., Metayer-Coustard S., Ji B., Rame C., Gespach C., Voy B., Simon J. (2012). Characterization of major elements of insulin signaling cascade in chicken adipose tissue: Apparent insulin refractoriness. Gen. Comp. Endocrinol..

[30] Proszkowiec-Weglarz M., Dupont J., Rideau N., Gespach C., Simon J., Porter T.E. (2017). Insulin immuno-neutralization decreases food intake in chickens without altering hypothalamic transcripts involved in food intake and metabolism. Poult. Sci..

