# Supplementary material for: Unrestricted Feed Intake Induces β-Cell Death and Impairs Insulin Secretion in Broiler Breeder Hens
Source: Animals (Basel). 2020 Oct 26;10(11):1969. doi: 10.3390/ani10111969 (PMC7693285; doi:10.3390/ani10111969)
Supplement: Supplementary file 1 [file animals-10-01969-s001.pdf]

Supplementary data

Supplementary Table S1. **Ingredient composition of the basal broiler breeder layer diets.**

**Ingredients**	**%, w/w**
Corn	66.9
Soybean meal (CP 45%)	22.2
Soybean Oil	1.67
Calcium carbonate (ground oyster shell)	6.69
Dicalcium phosphate	1.87
Salt	0.18
L-Lysine	0.1
DL-Metionine	0.1
Choline-Cl (70%)	0.09
Mineral premix[1]	0.1
Vitamin premix[2]	0.1
Copper sulfate	0.05
**Nutrient composition (calculated)**	
Total metabolizable energy (ME, kcal/kg)	2920
Crude protein (%)	16
Crude fat (%)	4.15
Calcium (%)	3.24
Total phosphorus	0.9
Available phosphorous	0.41

[1]; Mineral premix provided the following per kilogram of complete diet: Cu, 18 mg; I, 1 mg; Fe, 80 mg; Mn, 150 mg; Zn, 125 mg; and Se, 0.25 mg.

[2]; Vitamin premix provided the following per kilogram of complete diet: vitamin A, 10,000 IU all-trans retinol equivalent; vitamin D, 2,500 IU cholecalciferol equivalent; vitamin E, 100 IU R.R.R-α-tocopherol equivalent; vitamin K, 3 mg phylloquinone equivalent; vitamin B12, 0.03 mg cyanocobalamin equivalent; riboflavin, 8 mg riboflavin equivalent; niacin, 60 mg nicotinamide equivalent; pantothenate, 18 mg pantothenic acid equivalent; folate, 1 mg folic acid equuivalent; Vitamin B6, 6 mg pyridoxine equivalent; thiamine HCl, 3 mg; and biotin, 0.2 mg.

Supplementary Table S2. Recommended body weight and feed allowance of Arbor Acres Plus FF broiler breeder hens

Age (weeks)	Target body weight (g)	Feed (g/bird/day)	Weekly gain (g)
**23**	2630	115	170
**24**	2810	123	180
**25**	2960	129	150
**26**	3110	136	150
**27**	3210	146	100
**28**	3270	162	60
**29**	3300	162	30
**30**	3325	162	25
**31**	3345	162	20
**32**	3365	162	20
**33**	3385	162	20
**34**	3405	162	20
**35**	3420	162	15
**36**	3435	162	15
**37**	3450	161	15
**38**	3465	161	15
**39**	3480	161	15
**40**	3495	160	15
**41**	3510	160	15
**42**	3525	159	15
**43**	3540	159	15
**44**	3555	159	15
**45**	3570	158	15

Data are adapted from Arbor Acres Plus FF Parent Stock Performance Objectives provided by the company at website;
http://ap.aviagen.com/assets/Tech_Center/AA_Breeder_ParentStock//AAFF-PS-PO-EN-2016.pdf

Supplementary Table S3. Primers for quantitative real time polymerase chain reaction (qRT-PCR) analysis

Gene	Access number	Primer (5'-3')	Amplicon (bp)	Optimized primer concentration
**β-actin**	GI:211236	F: CTGATGGTCAGGTCATCACCATT<br>R: TACCCAAGAAAGATGGCTGGAA	78	300 nM
**Insulin**	V00416.1	F:CCTCTTCTGGCTCTCCTTGTCTT<br>R:-TGGTTGGCAGCTGCATAGC	58	900 nM

F: forward, R; reverse

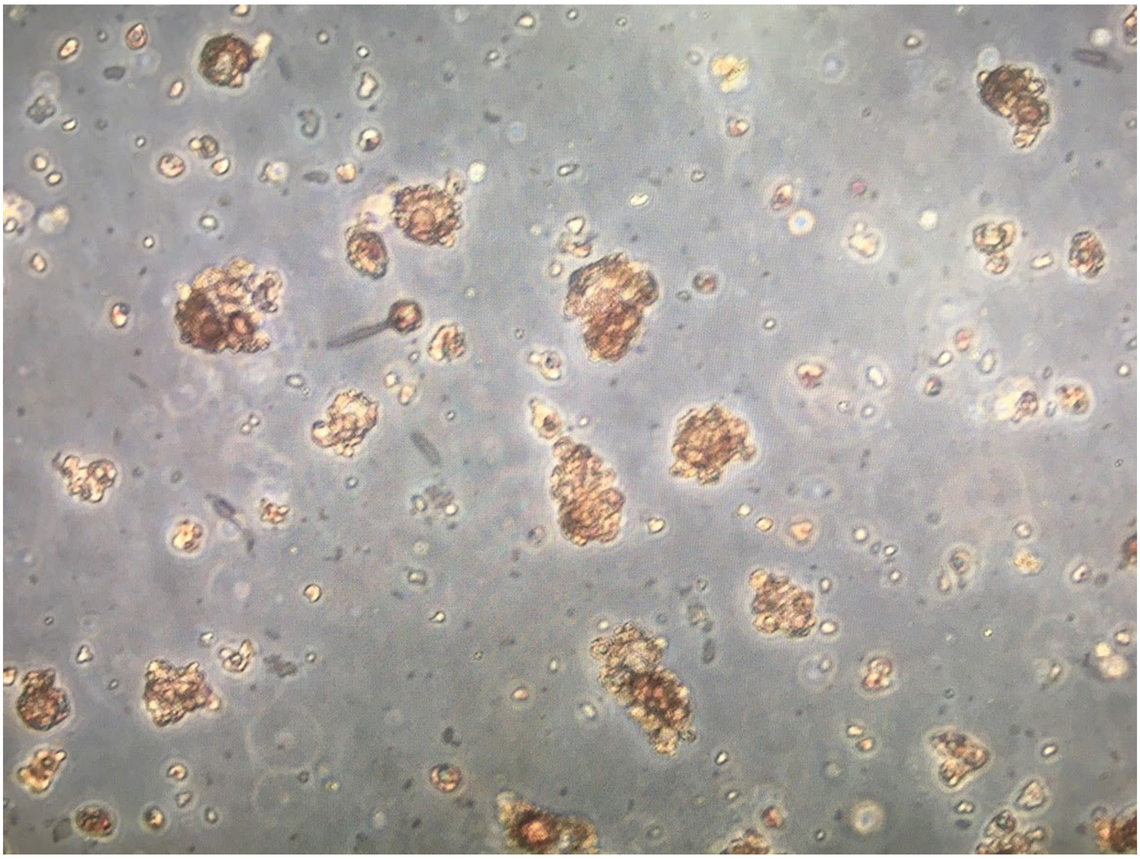

Supplementary Figure S1. Isolated β-islets of broiler breeder hens by diathizone staining.

An aliquot of isolated pancreatic β-islets was stained red by diathizone to confirm the purity under microscope examination (100×).

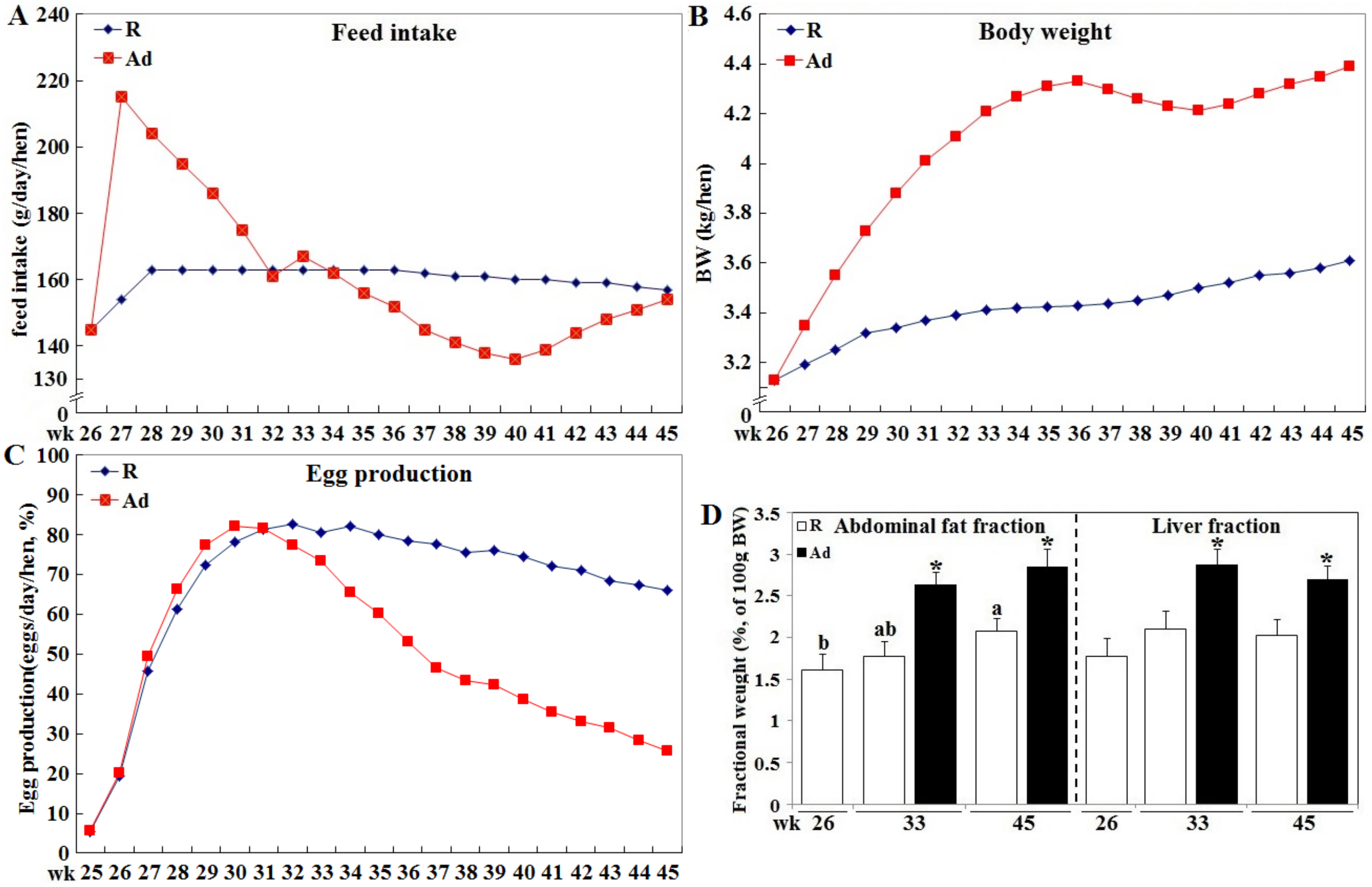

Supplementary Figure S2. Body weight, feed intake, egg production, and fractional liver and abdominal fat weight of broiler breeder hens provided with restricted or ad libitum feed intake.

At age of 26 wks, a half hens (n=70) of the flock was continued with breeder recommended restricted feeding protocol (Restricted group, R) and the remaining birds were allowed sufficient feed for consumption to appetite (Ad libitum group, Ad). Four hens at age of 26 wks under R-feed intake were sampled as a start control and thereafter 6 hens from both R- and Ad- group were necropsied at 33 and 45 wks for tissue collection and body composition analysis.

Means with different letters within the same feed intake are significantly different among times ($P < 0.05$).

*; significant difference by Ad-feed intake, $P<0.05$.

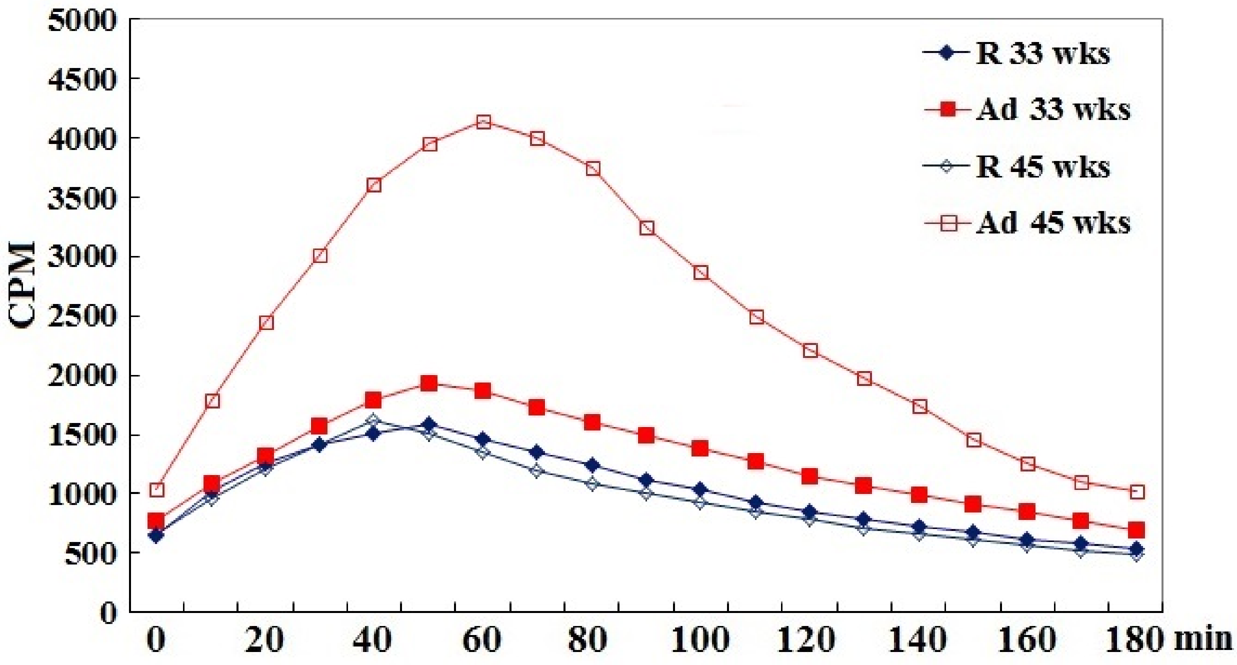

Supplementary Figure S3. Pancreatic β-islet caspase 3/7 activity of broiler breeder hens provided with restricted or ad libitum feed intake.

At age of 33 and 45 wks, 6 hens from each the R- and Ad-groups were randomly selected for necropsy and tissue collection. Isolated pancreatic β-islets were used for caspase 3/7 activity analysis using a fluorophoric peptide substrate.

R; restriction, Ad; ad libitum.
